# Monthly Summary

**Published:** 1872-04

**Authors:** 


### MONTHLY SUMMARY.

---

*Painless Extraction of Teeth.*—Dr. A. C. Castle, (*Dental Cosmos*) observes that he has for thirty years adopted the plan of obtunding or benumbing the extremities of the temporal nerves for painless extraction of teeth from their sockets, with complete success, never having used or countenanced the exhibition of chloroform, ether, or nitrous oxide gas for this minor surgical operation. The benumbing, or mechanical anæsthesia, of the temporal branches of nerves, obtunds the whole nerve to a sufficient extent to allow the teeth to be removed, with sensation so slight that, if not attending a special surgical operation, it would scarcely be noticed by the patient.

One of two modes may be adopted. By application of ice to the temples which is somewhat distressing, the sensation of cold striking deeply. The other, to which he gives the preference, is done by an assistant, with each of his middle fingers pressing with persistent firmness into the *fossa* or hollow behind the ridge of the temporal bone, which forms the external bone circle orbit of the eye, Pressure for one minute is all that is necessary. The practice is as simple as it is harmless, and leaves no after unpleasant sensation to annoy the patient. It is an instinctive method often adopted by people themselves, who press their temples to relieve themselves temporarily of the acute paroxysms of nervous headache. This temporary pressure, with sufficient force, is all that is required to remove teeth painlessly.

*External Application of Chloral Hydrate.*—Dr. Charles S. Strother, Barnesville, Ga., (*Atlanta Med. and Surg. Journal*), calls the attention of the profession to the external application of hydrate of chloral as a powerful and speedy counter-irritant, and, to a considerable extent, a local anodyne. The idea first suggested itself to Dr. McDowell in 1870, when he accidentally applied a small portion of chloral to his own face, instantly producing a burning sensation. It has been used with good results in facial neuralgia, pleurodynia, rheumatism, gastralgia, in uterine and ovarian pains, in advanced phthisis, and in relieving persistent nausea and vomiting. The mode of applying the chloral is as follows;—Place ℈ i. in a plate or a saucer. add water to made a saturated solution, and then apply with the tips of the fingers, with slight friction, over a space as large as the palm, or *pro re nata*. Should the burning be too intense, the parts may be sponged off in a few minutes with a cloth wrung out of warm water; and then apply glycerine with a little spirits camphor, or the glycerine alone, olive oil, or sweet cream.—*Med. News.*

---

*Treatment of Facial and Dental Neuralgia.*—This method consists in turning into the meatus auditorious from four to ten drops (according to the age and sensibility of the patient) of the following fluid; then to close the opening of the ear by means of a little cotton, and to cause the patient to hold the head inclined for some minutes to the side opposite to the seat of the pain, so that the liquid may remain in the bottom of the ear. This preparation is thus made:

℞—Ext. opii......................
Ext. belladonna ....... ......
Ext. stramonia............. .., *aa*, parts j.
Aq. pruni virg.............. . parts xij.
Solve et cola.

Although this preparation may be only extemporaneous, it may nevertheless be preserved, if care is taken to keep it cool, by pouring on its surface from two to four drops of sweet almond oil.

It is very rare, with the use of this liquid, that relief is not obtained in a few minutes, and the patient asleep in half an hour, whatever may have been the severity of the pains, and that without having been in the least danger. Absorption takes place almost as rapidly as from a denuded surface, and it is, therefore, unnecessary to blister the patient when we wish to use narcotics, since they act almost as rapidly by the auditory passage.

If it should happen that at the end of eight or ten minutes, the pain does not yield to the remedy—which sometimes happens when the quantity used has been too small, or when we have to treat a neuralgia which has already required the use of narcotics in any way—it is necessary to use the second dose, at least equal to the first, but in the opposite case, in order to obtain, promptly that relief which is only too frequently momentary, of facial neuralgias of long standing.—*Ibid.*—*Nashville Med. and Surg. Journal,*

---

*A Novel Surgical Operation* was recently performed by a San Francisco physician. The patient had been shot. The bullet entered the right side, a little above the hip, and in probing the wound the surgeon discovered where the bullet lay. He was compelled to enlarge the orifice of the wound in order to introduce the forceps, when the wounded man, who was under the influence of liquor, struck him a powerful blow on the side of the head, which caused him to fall to the floor. He then ran away, and when the doctor recovered himself, he found the forceps in one corner of the room and near the instrument was the bullet, which had been extracted by the force of the blow. The doctor says that as a surgical operation it was a complete success, but still he is not partial to the method.—*Medical and Surgical Reporter.*

---

*Chloral for Toothache.*—Dr Page, in the *British Med. Journal,* recommends chloral hydrate as a local application in cases of toothache. A few grains of the solid hydrate introduced into the cavity of the tooth upon the point of a quill speedily dissolves there; and in the course of a few minutes, during which a not unpleasant warm sensation is experienced, the pain is either deadened, or more often effectually allayed. A second or third application may be resorted to if necessary.

*Cell or Skin Grafting.*—Having had some experience in this exceedingly interesting practice, and having carried it farther than any of whose experiments I have read, I propose to give my "experience," or rather the results of my observations.

I have practised three methods: 1st, that of snipping off portions of true skin with the epithelial layer; 2nd, scraping off the epithelial layer; 3d, removing sheets of detached portions of epithelium, and transplanting these to the surface of ulcers not inclined to heal.

The first method is more tedious requires more care, and is less satisfactory than either of the others. As recommended, I took bits of skin about half the size of canary seeds, and these were carefully placed with the cut surface on the clean surface of the ulcer. To accomplish this, I take a fine cambric needle, fix in a handle, pass it through as small a piece of skin as I can, and then pass a sharp knife, with a sawing motion, under the needle, with the side of the knife closely pressing the needle, so as to cut the skin at the point where the deeper surface of the needle is in contact with in. I then lay the needle on the ulcer (with the graft upon it) in the same relative position it was upon the skin, dip the point of my knife in water, and placing its back at right angles upon the needles, draw the needle out from eye to point, thus sweeping the graft from the needle, and leaving it on the ulcerated surface. Then I apply a pretty thick layer of simple cerate on lint, and cover the surface with it. A pad of cotton wool, and finally a bandage smoothly applied, complete the process. Tne dressing is not changed for a week. At the end of this time it is probable no trace of the grafts can be recoguized; in one week more they will be apparent, and at the end of a month they will be found as large as the finger-nail.—*St. Louis Medical and Surgical Journal.*

---

*A New Styptic.*—Collodion 100 parts; carbolic acid, 10 parts; tannin (Pelouse's), 5 parts; benzoic acid (from gum), 5 parts. Mix the ingredients in the order above written, and agitate solutian is effected. This preparation has a brown color, and leaves on evaporation a strong adherent pellicle. It instantly coagulates blood, forming a consistent clot, and a wound rapidly cicatrizes under its protection.—*Med. and Surg. Journal.*

*White Gutta-Percha.*—The *Journal of Applied Chemistry* gives the following method of preparing this, for dentists' use and for other purposes. Four ounces of pure gutta-percha are digested with five pounds of methyl-chloroform until the solution is thin enough to pass through filtering paper. It is then filtered (an additional pound of chloroform will facilitate this), and should then be clear and nearly colorless. Alcohol is now added in sufficient quantity to precipitate the gutta-percha in a voluminous white mass, which is washed with alcohol, pressed in a cloth, and dried in the air. It must finally be boiled in water in a porcelain vessel for half an hour, and while still hot rolled into sticks. The chloroform can be separated from the alcohol by adding water, and the alcohol recovered by distillation.

---

*Styptic Cotton.*—In the September number of the London *Pharmaceutical Journal* for 1870 there is an extract from the *Schwabischer Merkur*, written by Dr. Ehrle, of Isny, in which he gives the following formula for the preparation of the cotton :

"Take cotton of the best quality ; boil in a weak solution of soda (four per cent.) about an hour ; wash with cold water ; press out and dry. Then steep the cotton in a solution of the chloride of iron (diluted one third) ; press and air dry; after which pick to pieces."

This prepared cotton is of a yellowish brown color, and requires to be protected from the air, as it absorbs moisture very rapidly, owing to the deliquescent character of the iron-salt which it contains. Having prepared some for experiment a few months ago, after using it for a time it occurred to me that if the sol. ferri subsulph. were used in its preparation instead of the sol. ferri chlor., a superior article would be made, inasmuch as the cotton would not then absorb moisture, and would not need the care to protect it which this did. I made some accordingly, and found it quite as efficient as a styptic, and entirely free from the objection referred to.

By the sample which I herewith send you, it will be seen that it is of a light straw color and perfectly dry to the touch. It is easily carried and ever ready for use. It acts chemically and mechanically—chemically through the iron-salt which it contains, and mechanically by means of its bulk and closely pressed fibres.

It has been in constant use at the St. Mary's Hospital for some months, and is looked upon by the surgeons there as a valuable addition to their armamentarium. The many instances in which its valuable aid may be called upon will suggest themselves to the surgeon and obstetricion, and need not, therefore, be specified here. While there is nothing new in presenting either of these iron-salts as a styptic, yet I think the profession indebted to Dr. Ehrle for the idea of putting them in a much more available and efficient form.—*Dr. James Cummiskey in Med. Times.*

---

